# RNA-seq from archival FFPE breast cancer samples: molecular pathway fidelity and novel discovery

**DOI:** 10.1186/s12920-019-0643-z

**Published:** 2019-12-19

**Authors:** Nathan D. Pennock, Sonali Jindal, Wesley Horton, Duanchen Sun, Jayasri Narasimhan, Lucia Carbone, Suzanne S. Fei, Robert Searles, Christina A. Harrington, Julja Burchard, Sheila Weinmann, Pepper Schedin, Zheng Xia

**Affiliations:** 1 Department of Cell, Developmental and Cancer Biology, Oregon Health & Science University, 2720 SW Moody Ave, Portland, OR 97201 USA; 2 Knight Cancer Institute, Oregon Health & Science University, 2720 SW Moody Ave, Portland, OR 97201 USA; 3 Computational Biology Program, Oregon Health & Science University, Portland, OR 97201 USA; 4 Division of Genetics, Oregon National Primate Research Center, Oregon Health and Science University, Beaverton, OR 97006 USA; 5 Department of Medicine, Knight Cardiovascular Institute, Oregon Health & Science University, 3303 SW Bond Ave, Portland, OR 97239 USA; 6 Bioinformatics & Biostatistics Core, Oregon National Primate Research Center, Oregon Health and Science University, Beaverton, OR 97006 USA; 7 Integrated Genomics Laboratory, Knight Cancer Institute, Oregon Health & Science University Knight Cancer Institute, Portland, OR 97239 USA; 8 Center for Health Research, Kaiser Permanente Northwest, Portland, OR 97278 USA; 9 Young Women’s Breast Cancer Translational Program, University of Colorado Anschutz Medical Campus, 1665 Aurora Court, USA, Aurora, CO 80045 USA; 10 Department of Molecular Microbiology and Immunology Oregon Health & Science University, Portland, OR 97273 USA

**Keywords:** FFPE, Formalin fixed paraffin embedded, Breast Cancer, RNA sequencing, RNA-Seq, genomics, Archival tissue, Regulon, RNA-seq, Estrogen receptor, KDM4B

## Abstract

**Background:**

Formalin-fixed, paraffin-embedded (FFPE) tissues for RNA-seq have advantages over fresh frozen tissue including abundance and availability, connection to rich clinical data, and association with patient outcomes. However, FFPE-derived RNA is highly degraded and chemically modified, which impacts its utility as a faithful source for biological inquiry.

**Methods:**

True archival FFPE breast cancer cases (*n* = 58), stored at room temperature for 2–23 years, were utilized to identify key steps in tissue selection, RNA isolation, and library choice. Gene expression fidelity was evaluated by comparing FFPE data to public data obtained from fresh tissues, and by employing single-gene, gene set and transcription network-based regulon analyses.

**Results:**

We report a single 10 μm section of breast tissue yields sufficient RNA for RNA-seq, and a relationship between RNA quality and block age that was not linear. We find single-gene analysis is limiting with FFPE tissues, while targeted gene set approaches effectively distinguish ER+ from ER- breast cancers. Novel utilization of regulon analysis identified the transcription factor KDM4B to associate with ER+ disease, with KDM4B regulon activity and gene expression having prognostic significance in an independent cohort of ER+ cases.

**Conclusion:**

Our results, which outline a robust FFPE-RNA-seq pipeline for broad use, support utilizing FFPE tissues to address key questions in the breast cancer field, including the delineation between indolent and life-threatening disease, biological stratification and molecular mechanisms of treatment resistance.

## Background

Within oncology, the ability to rapidly characterize a whole tumor transcriptome has resulted in the increasing stratification of patient cohorts by molecular subtype. These data inform disease diagnosis, selection of precision therapies, and prognosis. To date, the efforts to build molecular profiles of common malignancies, including The Cancer Genome Atlas (TCGA), utilized fresh tumor specimens, which provide high quality RNA analyses [1]. However, for any given cancer, only a small fraction of total cases have enough fresh tissue available for unbiased exosome-level RNA expression, and further, even fewer cases associate with long term outcomes data. For example, ER+ breast cancer has a 5 year survival rate of greater than 95%, but a 20–40% progression to metastasis over the course of 10–20 years [2–6]. To begin to address the barriers of limited outcomes data, one approach is to use formalin-fixed paraffin-embedded (FFPE) tissue for RNA sequencing, which has several advantages. First, biorepositories of FFPE tissue are maintained at all cancer care hospitals. Second, FFPE tissues can be linked to in-depth patient clinical data to create robust experimental and control groups. Third, FFPE tissue is generally archived for at least 10 years and can thus be associated with longer-term patient outcomes.

Despite the advantages of FFPE tissues, uncertainty about the fidelity of FFPE RNA remains a serious limitation. FFPE tissue processing and sample storage are known to result in highly degraded RNA, which limits gene detection and introduces sequencing artifacts [7, 8]. Nonetheless, groups have made important technical advances [9–12] in the analysis of patient FFPE tissues, resulting in the subdivision of patient cohorts into distinct molecular subtypes with prognostic significance [13–17]. Despite these important advances, concerns regarding data quality and interpretation remain, limiting the full potential of FFPE archival specimens to advance omics-based oncologic inquiry.

In this report, we define an effective FFPE-RNA-seq pipeline, identifying several key steps related to tissue selection, RNA isolation, library selection and data analysis. Using this optimized pipeline, we utilize true archival FFPE breast cancer (BrCa) tissues to distinguish between ER+ and ER- breast cancer cases, and confirm gene expression fidelity by comparing FFPE data to publicly available databases obtained from fresh tissues. Using novel analytical methodologies beyond single-gene comparisons, we identify key molecular pathways that distinguish ER+ and ER- breast cancers with high confidence. Further, in a proof-of-principle application, we identify the transcriptional regulator KDM4B to associate positively with active ER signaling and provide prognostic significance for patient outcomes.

## Methods

### Ethics approval and consent

The research was conducted on archived FFPE tissue samples collected under IRB-approved protocols at University of Colorado and Kaiser Permanente Center for Health Research. These tissue archives are comprised of clinical samples obtained from women with invasive cancer who were receiving standard of care. For this study, breast specimens from premenopausal women aged 20–45 years were obtained under IRB approvals (OHSU IRB# 00010989, # 15361). All cases were de-identified to the research team at all points and therefore this study was considered exempt for participation consent by the participating IRBs.

### Sample description

The obtained archival FFPE breast cancer (BrCa) tissues (*n* = 58) had been stored at room temperature between 2 and 23 years before RNA isolation was performed. For all cases, multiple H&E slides were reviewed from each case by a pathologist and sections with tumor were selected for inclusion in the study. Adjacent serial unstained sections were then submitted for RNA extraction.

### RNA isolation

Total RNA was extracted from recently cut 10 μm FFPE sections using the miRNeasy FFPE kit (Qiagen, Valencia, CA) according to the manufacturer’s protocol, using 1–4 sections (10–40 μm) per case depending on assay. RNA yield and quality were determined by UV absorption on a NanoDrop 1000 spectrophotometer and fragment size was analyzed using the RNA 6000 Nano assay (Agilent Technologies, Santa Clara, CA) run on the 2100 Bioanalyzer. DV200 values representing the percentage of RNA fragments above 200 nucleotides in length were determined according to Agilent and Illumina recommended protocols [18]. To determine the minimal amount of tissue needed to yield adequate RNA quantity for library preparation, RNA yield per 10 μm section number was tested. Based on the test results, one or two 10 μm sections of breast FFPE specimens were used for RNA isolation. RNA quality was assessed using DV200 values and cases with DV200 more than 27% were included for library preparation.

### Library preparation and sequencing

Two library preparation methods were tested. An input of 75 ng of total FFPE RNA was used with the TruSeq RNA Access Library Prep Kit (Illumina, San Diego, CA) and an input of 150 ng of total FFPE RNA was used with the Ovation Human FFPE RNA-seq Library System (NuGEN Technologies, San Carlos, CA). Libraries were prepared in triplicate from two FFPE RNA samples according to manufacturer instructions (3 technical replicates for each sample and method, Table 1). Libraries were quantified by real-time PCR using KAPA Library Quantification kits (Kapa Biosystems, Wilmington, MA) on ABI StepOne thermocycler, pooled according to library method (6 libraries per lane), and sequenced on a Hi-Seq 2500 (Illumina) using a 100 cycle, single end protocol providing approximately 45 million reads per sample. Base call files were converted to fastq format using Bcl2Fastq (Illumina). For the studies interrogating ER+ and ER- breast cancers, library preparations were performed using only the TruSeq RNA Access protocol with 75 ng RNA input. RNA-seq libraries of six samples, ER+ (*n* = 3) and ER- (*n* = 3), were generated from FFPE RNA with DV200 values ranging from 27 to 44%. Libraries were quantified as described and pooled at 3 libraries per lane. Sequencing was performed on a Hi-Seq 2500 using a 100 cycle, single read protocol with a depth of approximately 90 million reads per sample. Following initial sequencing, 3 of the 6 libraries were repooled and independently sequenced. Base call files were converted to fastq format using Bcl2Fastq (Illumina).

**Table 1:** RNA-seq Sample Characteristics

Figure: Sample	Year of Diagnosis	ER Status - IHC	Sample Type	GEO- Accession #
Figure 2: S1	2009	Positive	FFPE	GSM3737461, GSM3737462, GSM3737463,GSM3737467, GSM3737468, GSM3737469
Figure 2: S2	2010	Positive	FFPE	GSM3737464, GSM3737465, GSM3737466, GSM3737470, GSM3737471, GSM3737472
Figure 3-6: S1	2002	Positive	FFPE	GSM3737473, GSM3737474
Figure 3-6: S2	2005	Positive	FFPE	GSM3737475, GSM3737476
Figure 3-6: S3	2010	Positive	FFPE	GSM3737477
Figure 3-6: S4	1997	Negative	FFPE	GSM3737478
Figure 3-6: S5	2011	Negative	FFPE	GSM3737479
Figure 3-6: S6	2009	Negative	FFPE	GSM3737480, GSM3737481
Figure 3-6: P1	2000	Positive	Fresh-Frozen	GSM1401676
Figure 3-6: P2	2001	Positive	Fresh-Frozen	GSM1401677
Figure 3-6: P3	2001	Positive	Fresh-Frozen	GSM1401678
Figure 3-6: P4	2001	Positive	Fresh-Frozen	GSM1401679
Figure 3-6: P5	2001	Positive	Fresh-Frozen	GSM1401680
Figure 3-6: P6	2001	Positive	Fresh-Frozen	GSM1401684
Figure 3-6: P7	2009	Positive	Fresh-Frozen	GSM1401713
Figure 3-6: P8	2010	Positive	Fresh-Frozen	GSM1401715
Figure 3-6: P9	2002	Positive	Fresh-Frozen	GSM1401716
Figure 3-6: P10	2006	Positive	Fresh-Frozen	GSM1401717
Figure 3-6: P11	2005	Negative	Fresh-Frozen	GSM1401719
Figure 3-6: P12	2006	Negative	Fresh-Frozen	GSM1401720
Figure 3-6: P13	2003	Negative	Fresh-Frozen	GSM1401721
Figure 3-6: P14	2002	Negative	Fresh-Frozen	GSM1401722
Figure 3-6: P15	2004	Negative	Fresh-Frozen	GSM1401724
Figure 3-6: P16	2001	Negative	Fresh-Frozen	GSM1401726
Figure 3-6: P17	2009	Negative	Fresh-Frozen	GSM1401727
Figure 3-6: P18	2010	Negative	Fresh-Frozen	GSM1401729
Figure 3-6: P19	2001	Negative	Fresh-Frozen	GSM1401733
Figure 3-6: P20	2004	Negative	Fresh-Frozen	GSM1401757

Each sample designation is identified with relevant figures. Year of diagnosis indicates the year the specimen was collected. ER status in all cases was performed by clinical diagnostic IHC. Sample type for retrieval of RNA is indicated as either Formalin Fixed Paraffin Embedded (FFPE) or fresh tissue processed and stored frozen (Fresh-frozen). GEO Accession number for ascribed samples is indicated

### Public data

A total of 20 fresh samples (10 ER+ and 10 ER-) were selected from Varley et al. [19] (GEO accession GSE58135, Table 1, P1–20).

### RNA sequence alignment

All RNA-seq reads were aligned to the human reference genome (GRCh38, release 84) using STAR (version 2.5.2b) [20]. The STAR “GeneCounts” module was used for gene quantification, with the resulting strand counts chosen depending upon library preparation (Access – reverse; Ovation – forward; public – unstranded). Soft-clipping and mismatch tolerance were modified to evaluate the best possible alignment (see results), with default parameters ultimately chosen.

### Data processing and significance testing

STAR read counts were used as input into DESeq2 [21]. Genes with counts per million (cpm) greater than 0.05 in three or more cases were kept for subsequent differential expression gene (DEG) analyses. DEG analysis was performed with ER status as the variable of interest and DEG were called based upon a false discovery rate (FDR) less than 0.05. A log 2 fold-change threshold of 1 was also set. After normalization analyses, counts were transformed using the variance-stabilizing transformation (VST) module in DESeq2 for downstream analyses.

### Access vs. Ovation library comparison

Counts output by STAR were normalized using a variety of different methods. Genes with Access-identified probes and counts per million (cpm) greater than 1 in 50% of samples from at least one library preparation method (14,432 genes) were selected for visualizations.

ER+ vs. ER-: Genes with Access-identified probes and counts per million (cpm) greater than 1 in 50% of either FFPE or fresh-frozen samples (14,331 genes) were selected for analysis. Differential expression analysis was performed using DESeq2 [21] with ER status as the factor of interest. Counts adjusted via the variance-stabilizing transformation were utilized for subsequent visualizations.

### Sample-to-sample distances

Euclidean distance, using ‘dist’ in R, was calculated based on the VST gene expressions to produce an aggregate sample-to-sample distance matrix. To assess global similarity of FFPE and fresh-frozen gene expression profiles, pairwise Pearson correlations between all samples were visualized using the ‘pheatmap’ R package [22]. Dendrograms were created using complete clustering of the Euclidean distances between the resulting correlations. Additionally, Principal Component Analysis (PCA) was also performed on all genes using the ‘plotPCA’ function of the DESeq2 package, with ER status as the variable of interest. Principal component analysis (PCA) was performed on VST gene expression values using default parameters. Non-negative matrix factorization (NMF) clustering was used to cluster the original and resequenced samples, using all genes output by DESeq2. VST gene expressions were filtered for top 100 differentially expressed (DE) genes or defined genes ranked by adjusted *p*-values. Filtered genes were plotted using ‘pheatmap’ [22] and samples were clustered using default Euclidean distance.

### BrCa subtype prediction

Tumor biologic subtypes (Luminal A, Luminal B, Basal, HER2) were predicted using the PAM50 prediction parameters as determined by Parker et al. [23].

### Gene selection for fresh vs. FFPE comparison

We subset FFPE samples for protein-coding genes with a cpm greater than 1 in at least 3 cases, resulting in 13,807 genes. Fresh samples were filtered for these exact genes. All data were combined together and run through DESeq2 with ER status as the variable of interest with a log 2 fold-change threshold of 1, as well as kept separately (e.g. all fresh samples only) and subjected to the same analysis. In addition, all ER+ cases were grouped together and DESeq2 was performed with sample type as the variable of interest, with the same done for ER- cases.

### Cancer set Heatmaps

To assess the ability of previously reported cancer gene sets to distinguished cohorts, transformed counts were subset for all matching genes from the Oncotype DX, MammaPrint, and PAM50 gene sets. Gene expression data were z-score transformed for each gene for visualization purposes. Dendrograms were produced using complete clustering of the pairwise Pearson correlation values. Pearson correlation was calculated on raw values for the Oncotype DX and MammaPrint subsets, and z-score transformed values for the PAM50 subset, in accordance with the methodology of each gene set’s construction. For PAM50, subtypes as well as proliferation, ER, and HER2 scores were generated using the original prediction parameters determined by Parker et al. [23].

### Gene set enrichment analysis (GSEA)

GSEA version 3.0 was used to identify gene sets from the Hallmark database (v6.1, ‘h.all.v6.1.symbols.gmt’) as well as custom gene sets (described below) that were significantly enriched between ER+ and ER- cases or between sample storage methods. When testing for differences between ER status, FFPE and fresh-frozen samples were individually divided by ER phenotype and queried against reference sets. When testing for differences between sample storage methods, all samples were divided by storage method and queried against reference sets. Custom gene sets were created by determining genes that were unique or commonly identified as statistically significant (*p* ≤ 0.05, Fisher’s exact test using all input genes (14,330) as background results in *p* < 2.2e-16**)** differentially expressed genes between ER+ (red) and ER- (blue) samples from FFPE or fresh specimens (Fig. 5).

### Regulon analysis

We used the master regulator inference algorithm (MARINa) [24] compiled in R ‘viper’ package [25] to perform the regulon analyses on breast cancers from FFPE tissue and the publicly available fresh-frozen datasets, respectively. Two sources of data, gene expression signature and regulatory network, were required as model inputs. In this work, the Wald test statistics in DESeq2 that quantify the difference of ER+ and ER- were used as gene expression signatures. As for regulatory network, we directly used the published breast cancer regulon network ‘regulonbrca’ curated in R package ‘aracne.networks’ that was reverse-engineered by ARACNe [25, 26] using TCGA breast cancer data [27].

The single-sample-based regulon activities were inferred by function ‘viper’, which is an extension of MARINa [24] and transforms a gene expression matrix to a regulatory protein activity matrix. For the model input, we used the fragments per kilobase of transcript per million (FPKM) quantification of breast cancer samples in TCGA as the expression matrix and the same regulon network ‘regulonbrca’ as the regulatory network.

### TCGA – Breast Cancer Kaplan-Meier analysis

The key genes/transcription factors determined from BrCa regulon analyses were selected as genes of interest for determination of gene expression or regulon activity correlation with survival outcome. Kaplan-Meier plots were produced by downloading TCGA outcomes, cohort metadata and gene expression data through the UCSC-Xena Functional Genomics Browser [28] tool, which were then plotted in GraphPad Prism Software (v7.05, La Jolla California, USA) and the numbers of samples indicated in brackets for gene expression Hi (red) and Lo (blue) cohorts with *p*-values displayed determined by log-rank analysis.

## Results

### Assessing the amount of FFPE tissue needed for adequate RNA extraction

**Fig. 1:**
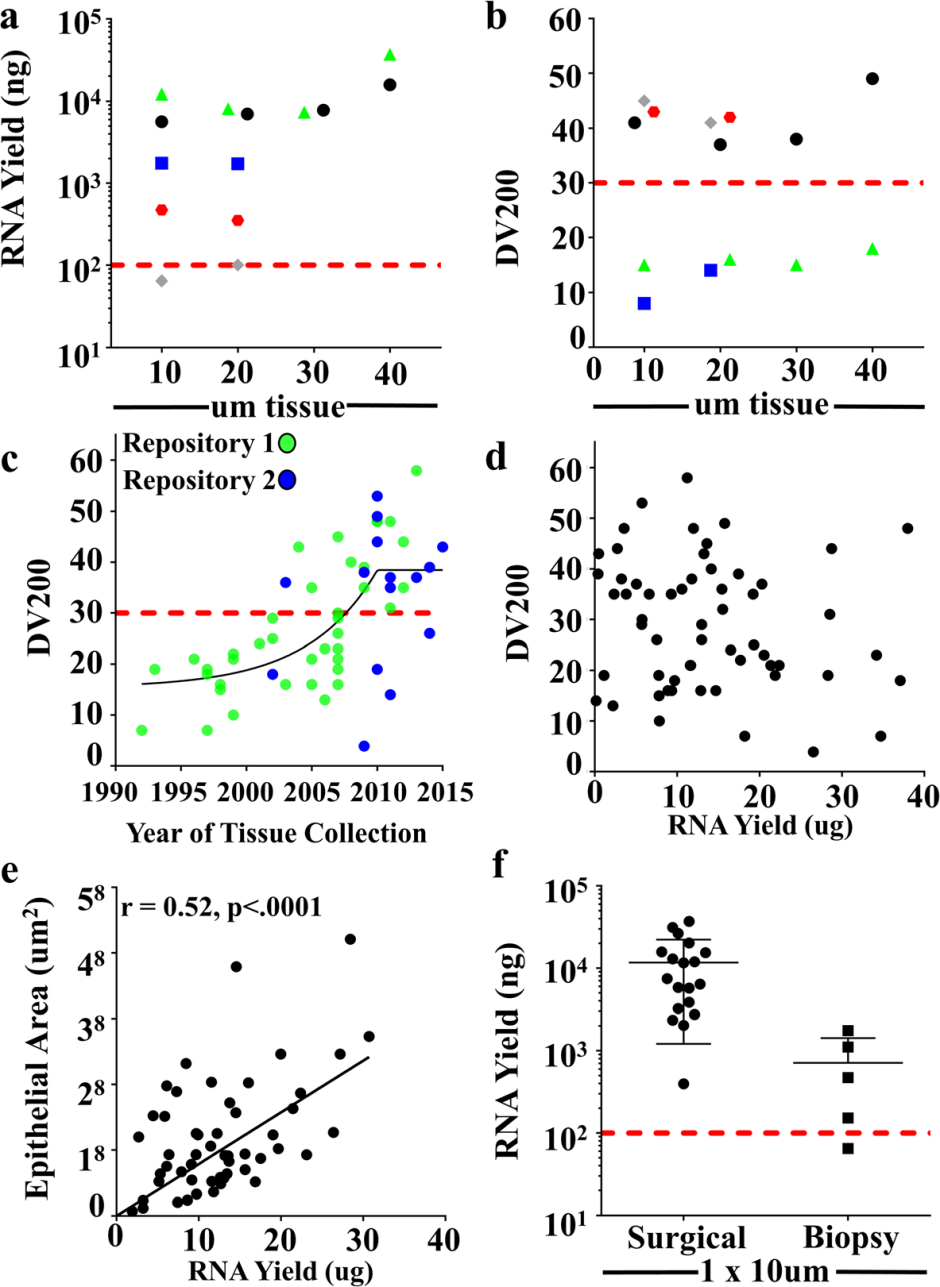
RNA quantity and quality are impacted by epithelial area and block age. **a**) Five separate archival FFPE blocks (each a different color and symbol) of breast cancer tissue were subjected to RNA isolation from 10 to 40 μm of tissue and evaluated for overall RNA yield and **b)** RNA quality determined by DV200 value. **c)** 58 archival breast cancer tissues from two different repositories (indicated by green and blue dots) were subjected to RNA isolation from one 10 μm section and evaluated for the relationship between DV200 and year of collection, (black line is best fit line using a centered fourth order polynomial) and **d)** overall RNA yield. **e)** Epithelial area and **f)** specimen type compared to yield. Red lines at 100 ng and DV200 = 30 denote recommended minimal technical limits for successful sequencing

While FFPE tissues are more abundant than fresh tumor tissue, these archival tissues still represent a limited resource that require judicious utilization. Thus, we initially set out to determine the minimum amount of FFPE breast tissue that will yield the quality and quantity of RNA needed for successful sequencing. Many protocols developed for extracting RNA from FFPE blocks suggest utilizing 40 micrometers (μm) of tissue, an amount that is 10 times greater than what is required for immunohistochemistry (IHC) evaluation. We first examined the influence of the number of 10 μm sections on overall RNA yield. 1, 2, 3 or 4 10 μm serial sections were used to isolate RNA from 5 separate FFPE breast cancer cases (Fig. 1a). In general, sufficient yield was obtained with a single 10 μm section, with no increased yield observed with the inclusion of additional sections. These observations strongly suggest that RNA isolation is saturated in capacity to capture RNA at 10 μm of FFPE breast tissue. Further, in 4 of 5 cases evaluated, the amount of RNA from a single 10 μm section was in vast excess of the 100 ng required for RNA sequencing (Fig. 1a, 100 ng level denoted by red line).

We next evaluated the quality of the isolated RNA as assessed by DV200 value. The DV200 value reports the percentage of purified RNA with length greater than or equal to 200 nucleotide bases. As a useful predictor of successful sequencing results, a DV200 value of greater than or equal to 30% (Fig. 1b, red line) is recommended by manufacturers for FFPE library preparations. Of these 5 test cases, only 3 had RNA of sufficient quality required for RNA sequencing. Further, inclusion of additional sections had no impact on DV200 value (Fig. 1b).

To investigate the impact of FFPE processing on the intra-case variability of RNA yield and quality, serial 10 μm sections of 6 cases were processed in 2 independent runs. Analysis revealed consistent inter-run values with ≤ 8% variation, data consistent with FFPE tissues being a reproducible source for RNA material (Additional file 1: Figure S1a and b). We next ascertained the relationship between age of block and RNA quality using 58 cases of breast cancer FFPE samples collected between 1992 and 2014. We found a drop in quality in cases greater than 11 years old (cases diagnosed before 2006) (Fig. 1c), with only ~ 13% (3 of 23) of these older cases having a DV200 ≥ 30% (Fig. 1c, red line). However, for the blocks ≤ 10 years of age, 82% (18 of 22) of cases had DV200 values greater than 30%. Further, we found evidence for possible variation in quality due to the specific institutional repository (green vs. blue circles), emphasizing the need to use empirical determination for block quality rather than relying on simple age metrics. Further, in this larger data set of 58 cases, we confirmed lack of correlation between DV200 and RNA yield (Fig. 1d).

To examine if RNA yield correlates with cellular composition (tumor area), sections were quantified for epithelial tumor content. A clear positive relationship emerged (Fig. 1e), with cellular content being the dominant determinant of RNA yield. Given this, we assessed the utility of extracting RNA from 10 μm biopsy sections compared to 10 -μm sections of surgically excised tissue, as utilization of biopsy material could greatly expand patient numbers and scope of research questions compared to surgical excision alone. Of the 5 biopsy samples with high DV200 (≥ 30%) all samples except one provided ≥100 ng of RNA required for advancement to sequencing (Fig. 1f, red line). In sum, these analyses confirm that single 10 μm FFPE sections of biopsies or surgical samples are suitable for advancing to RNA-sequencing.

### Library Preparation & Data Normalization

Given the highly fragmented and chemically modified properties of the input RNA from FFPE sources, we next evaluated the impact of library preparation on resulting gene expression profiles. We tested Illumina-Access and NuGEN–Ovation platforms, both useful library preparation methods for FFPE samples, which differ in how RNA is enriched and amplified. The Access kit is based upon biased, selective hybridization and enrichment of RNA using bead-conjugated oligomers designed for 19,000+ genes with > 10 different probes per gene. In contrast, the Ovation platform adopts an unbiased targeting approach utilizing both Poly A and random primer hybridization, resulting in a product with reduced ribosomal content and a more highly diverse RNA transcript library.

**Fig. 2:**
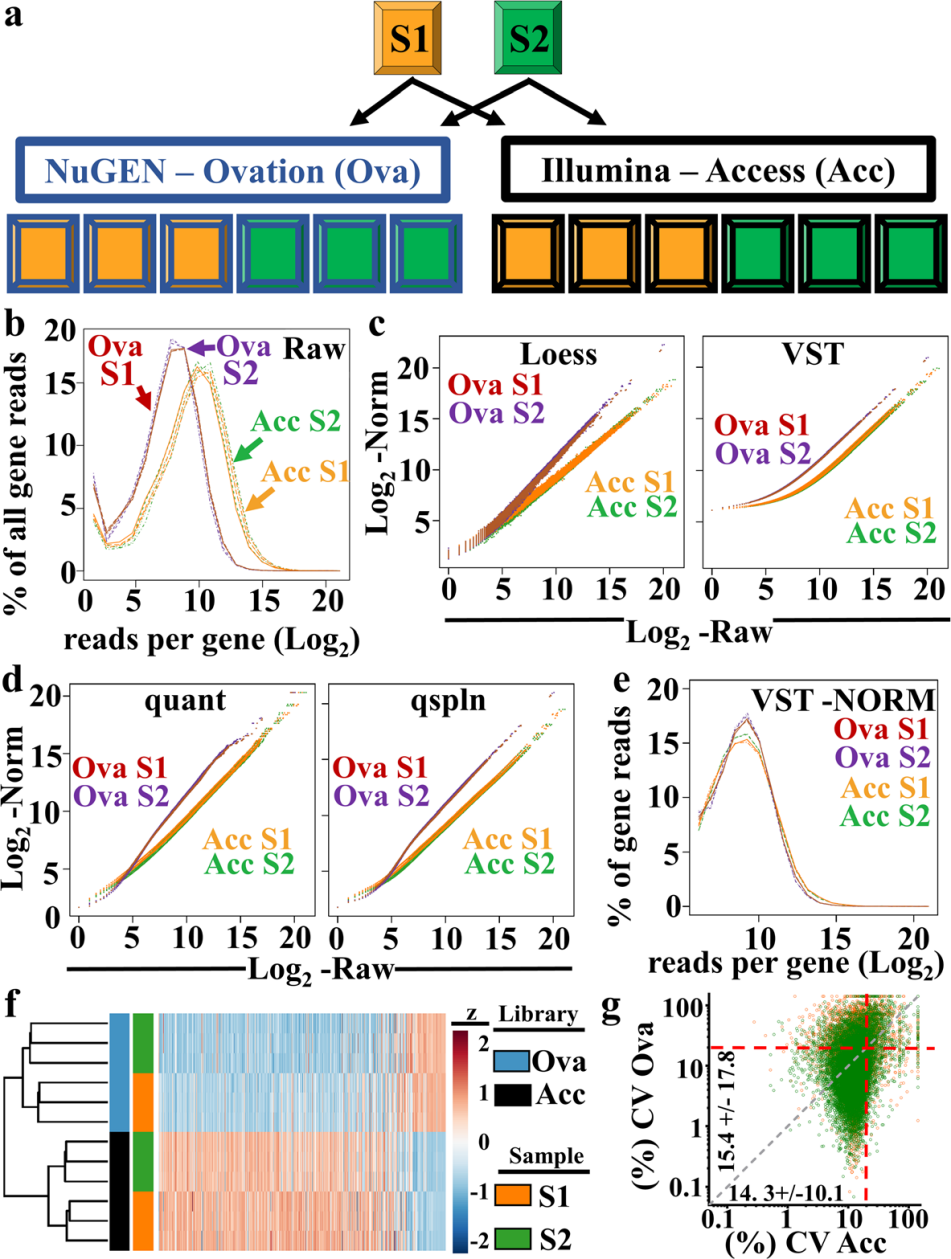
Evaluation of FFPE library preparations and data normalization approaches. **a**) Two FFPE samples (S1-orange, S2-green) were each prepared using two different commercial library preparations (NuGEN-Ovation-blue, Illumina-Access-black) in triplicate and sequenced by a Illumina Hi-Seq 2500 sequencer. Disparate library methodologies (Ovation (Ova-red and purple)), Access (Acc-orange and green)) resulted in clear differences in **b)** average raw counts per gene. Results of both libraries were subjected separately to **c)** Loess and VST, and **d)** quantile and q-spline data normalization approaches. VST normalization was selected based upon equal performance in both libraries across the range of reads and demonstrated success in **e)** normalization of average counts per gene in both libraries. **f)** Unsupervised hierarchical clustering of (14,432) normalized gene counts from S1 and S2 samples in triplicate reveals highest level of sample separation based upon library preparation (blue and black), followed by sample source. The 500 genes with the highest overall mean expression are shown. **g)** Library-based variation was assessed by plotting the coefficient of variation (CV) from triplicate samples for each gene for each library preparation (S1-orange, S2-green). Average % CV and standard deviation of CV is presented along corresponding axes. 20% CV is indicated by red dashed lines.

To compare these two library preparation platforms, two separate ER+ breast cancer FFPE biological samples (S1 and S2), each with a DV200 value > 30% were run in triplicate using both library preparation platforms (Fig. 2a). Sequencing reads from the Access kit provided higher counts per gene (Fig. 2b), but fewer relative numbers of unique reads compared to the Ovation kit (Additional file 1: Figure S1c and d). The Access kit data also lacked entire gene families, including mitochondrial RNA. We reasoned this loss occurred as a result of the Access kit being hybridization-based to oligos selected a priori for the included genes. For subsequent comparative analyses related to platform sensitivity and consistency, we focused on genes detected by both platforms, and next sought to compare read normalization approaches.

We compared 5 distinct normalization approaches, and the importance of considering normalization methodologies became apparent. Some methodologies, i.e., Loess, VST (Fig. 2c) and Limma (Additional file 1: Figure S1e and f) maintained the linear relationship between the raw and transformed values in both library platforms, while other approaches, i.e., quantile and q-spline, distorted raw values to normalized values based upon library methodology (Fig. 2d). Of the normalization approaches that produced tight and linear normalization to raw values, we elected to use VST normalization, as it is widely utilized as part of the DESeq2 analysis package where additional analytical tools are available. Using VST normalization, we confirmed comparable distributions of reads per gene for each of the 2 library preparation methodologies (Fig. 2e), highlighting the suitability of VST normalization for library performance comparison.

Next we performed unsupervised hierarchical clustering of these VST normalized gene counts and showed Illumina-Access and NuGEN–Ovation platforms clustered sample repeats together (S1-orange vs. S2-green) (Fig. 2f). Importantly, the first branch of the clustering dendrogram is driven by library preparation platform (blue vs. black), demonstrating that library preparation is a greater driver of gene expression differences than sample identity (S1 vs. S2). Given the aforementioned differences in library construction, this result is not surprising.

Having demonstrated the utility of both platforms to generate reproducible gene expression data from FFPE samples, we assessed which platform is most beneficial for our questions related to distinguishing differential gene expression between study groups (in this case, between S1 and S2). For a gene to be differentially expressed between groups, both the change in the magnitude and the degree of variability in gene expression need to be considered. Our experimental design of 2 independent samples assayed in triplicate is well suited for determining relative library variability. By plotting the coefficient of variation for each gene from one platform compared to the other, we observed that the Access platform displayed reduced variability with 11.1% of all genes examined with a coefficient of variation (CV) of greater than 20% (Fig. 2g, red dashed lines), compared to 23.2% in the Ovation platform. Less variance combined with higher raw read counts lead us to select the Access library platform for our subsequent FFPE RNA-seq pipeline optimization.

### Alignment parameter optimization for FFPE RNA-seq

**Fig. 3:**
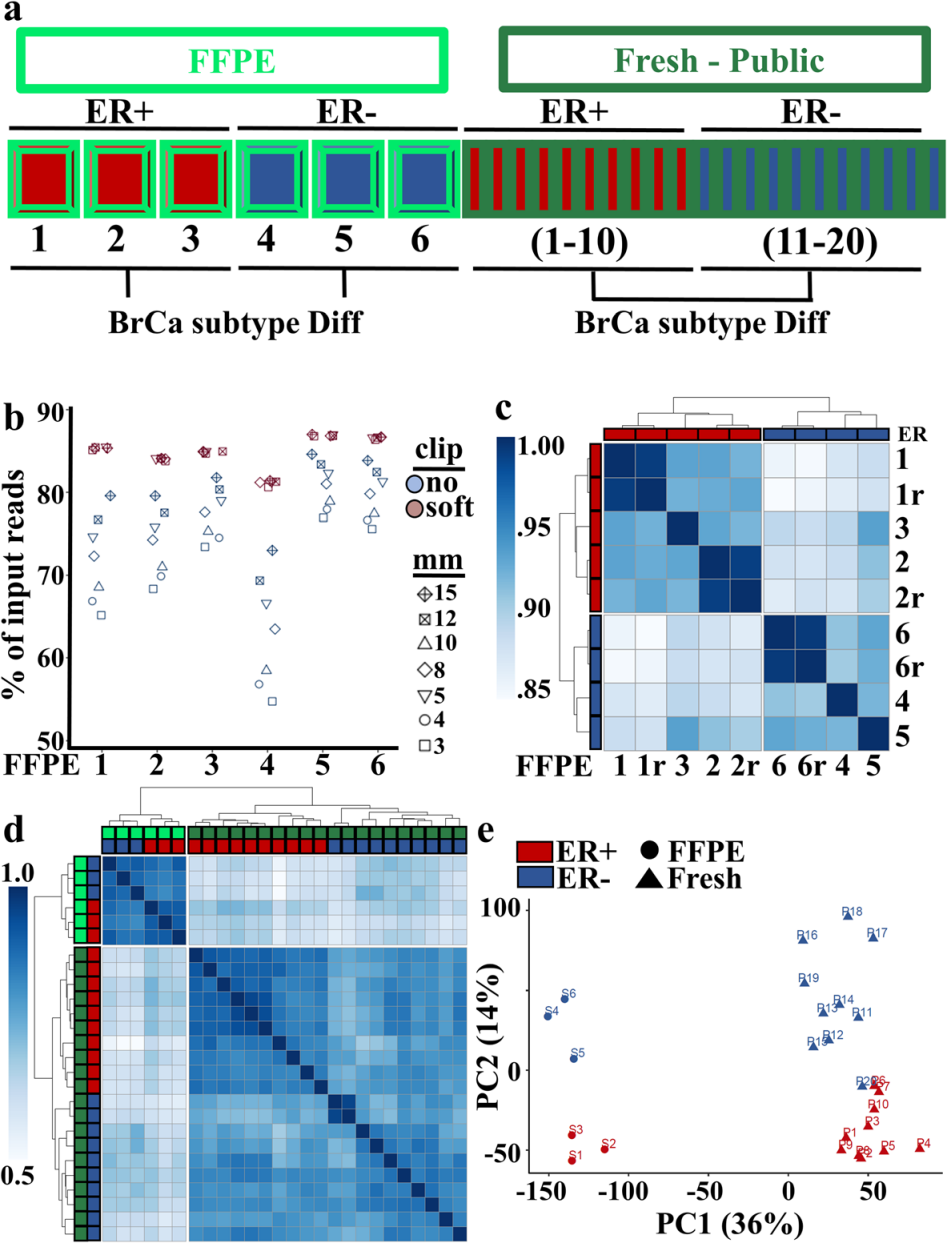
Breast cancer subtype identification of ER+ and ER- cases in global RNA-seq from FFPE and Fresh specimens. **a** Schematic of experimental design. RNA expression from ER+ (*n* = 3, red) and ER- (*n* = 3, blue) breast cancers from FFPE (bright green) specimens were compared to clinically verified ER+ (*n* = 10, red) and ER- (*n* = 10, blue) cases from a publicly available data set derived from fresh (dark green) tissue specimens. **b** Sequence alignment parameters of clipping (no = light blue, soft = light purple) and allowed nucleotide mismatches (mm) of 3–15 were evaluated for the 6 FFPE samples. **c** Unsupervised clustering based upon Pearson correlation values determined from global gene expression in 6 original FFPE samples and 3 samples whose libraries were resequenced (r) showing tight clustering of repeats and originals and clustering based upon ER status. **d** Unsupervised clustering based upon Pearson correlation plot of global gene expression correlation in 6 FFPE samples (light green) and 20 fresh samples (dark green) with clear separations firstly based upon sample type (FFPE vs. Fresh) and secondly based upon ER status (ER+ = red, ER- = blue). **e** Principal component analysis of the 6 FFPE (circle) and 20 Fresh (triangle) samples confirms separation based upon sample type (PC1) and ER status (PC2)

FFPE processing results in fragmented RNA and chemically altered residues that can lead to sequence alterations and read misalignment, leading to reduced aligned read counts [8, 29]. In an attempt to mitigate the impact of these artifacts, we addressed approaches to positively impact the number of mapped reads from FFPE-derived sequences to the reference genome. For these analyses, we used FFPE-derived sequences obtained from 3 ER+ [1–3] and 3 ER- [4–6] breast cancer cases, and compared soft-clipping as well as various mismatch allowances (Fig. 3a, left panel). For mismatch allowance, we adjusted mismatch parameters to increase the permitted number of imperfect nucleic acid residue alignments, which as anticipated, progressively increased the number of successfully aligned sequences (Fig. 3b, blue shapes). We then examined utilization of the soft-clipping approach, which ignores regions of residues on either side of the read that do not match well to the reference genome. In our FFPE sequence data, soft-clipping resulted in greater alignment than permissive mismatch parameters. Further, improvement in read alignment was unaffected by the addition of mismatched bases (Fig. 3b) These results suggest that a limitation to successful alignment of reads from FFPE RNA is end of read sequence quality, which soft-clipping accommodates, rather than intra-sequence nucleotide mismatch that has been noted to occur as an artifact of formalin fixation [30]. Based on these results, we proceeded with the use of the STAR alignment tool utilizing default parameters that include soft-clipping and 10 maximum mismatches for subsequent analyses of both FFPE and fresh tissue-derived expression data (Additional file 2: Figure S2a).

### Approach to evaluate pathway Fidelity in FFPE RNA-seq

We next examined whether sequence gathered from FFPE tissue recapitulates the gene expression and biological pathways determined from sequencing fresh tissue. Breast cancer subtypes have well-annotated RNA expression patterns based on ER expression [31], making breast cancer ideal for evaluating the fidelity of gene expression and pathway analysis in FFPE specimens. For this analysis, we first evaluated the reproducibility of genomic signatures derived from FFPE tissue by performing library resequencing of half of the samples (2 of 3 ER+ cases and 1 of 3 ER- cases). This approach allowed us to evaluate the strength of delineation between ER+ and ER- samples relative to the intra-specimen variation observed by resequencing. We observed the resequenced (r) sample libraries, which ran on different sequencing lanes and on different days, clustered with their original sample (Fig. 3c i.e., compare 1 and 1r, 2 and 2r, 6 and 6r). Secondly, RNA expression data from FFPE specimens perfectly segregated by ER status (blue vs. red, Fig. 3c). We next compared these FFPE results with public data derived from fresh samples (Fig. 3d). The most distinct separation occurred between the fresh and FFPE samples (light vs. dark green). However, within both fresh and FFPE cohorts we observed perfect clustering based upon ER status (Fig. 3d). When we employed principal component analysis (PCA) to understand what is driving the differences between fresh and FFPE samples, (Fig. 3e) we found the largest drivers were sample type variance (PC1), followed by ER status (PC2). While these broad evaluations of gene expression illustrated distinct differences between FFPE and fresh samples, evaluation of the mean value of a given gene revealed strong linear correlations between FFPE and fresh samples (Additional file 2: Figure S2b).

### Sample subtype separation by established gene panels

**Fig. 4:**
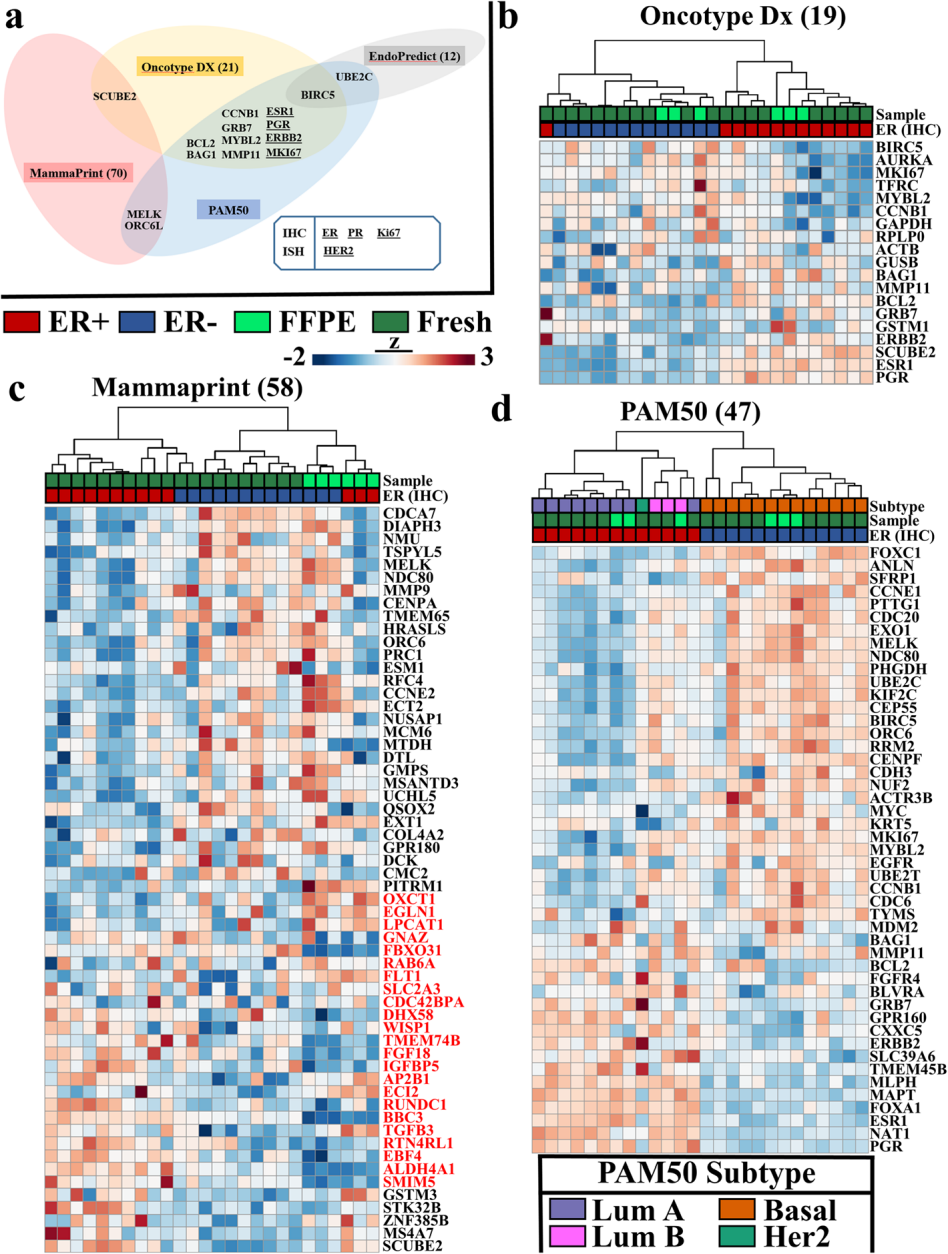
Breast cancer subtype identification in FFPE samples utilizing clinically relevant gene panels. **a**) Schematic of overlapping target genes in four clinically relevant gene panels; Oncotype DX, Mammaprint, Endopredict and PAM50. Size of gene panel is indicated in parentheses and genes whose protein products (IHC) or DNA amplification (ISH) are evaluated by clinical histological examination are underlined. Unsupervised hierarchical Pearson correlation clustering based upon RNA-seq gene expression values for targeted expressed genes from **b)** Oncotype Dx, **c)** Mammaprint and **d)** PAM50. ER+ cases are indicated in red and ER- in blue for both FFPE (light green) and Fresh (dark green specimens). Breast cancer intrinsic subtype assessment from PAM50 gene signature is displayed as Luminal A-dark purple, Luminal B-pink, Basal-orange and HER2-teal. PAM50 gene expression values were z-score transformed prior to clustering in alignment with protocols for subtype allocation

Another approach to compare gene expression fidelity of FFPE compared to fresh frozen tissues is to utilize the clinically relevant gene panels developed for breast cancer—namely, Oncotype Dx [14, 32], MammaPrint [33–35] and PAM50 [4, 23]. As shown (Fig. 4a), the gene composition of these three assays [36, 37] demonstrate very little overlap with one another, implicating each gene list as an independent opportunity to evaluate correlative performance between FFPE and fresh samples. In both the Oncotype Dx (Fig. 4b) and PAM50 (Fig. 4d) gene sets, the clustering of all samples was driven by ER status rather than sample preservation, with one imperfect ER clustering assignment observed for a fresh sample within the Oncotype Dx gene set. This demonstrates that the FFPE expression profiles look the most like fresh samples within the confines of these well-vetted gene sets. With MammaPrint, samples segregated largely by sample preparation type and secondarily by ER status (Fig. 4c). This was driven by loss of differential gene expression patterns in the FFPE samples, specifically in the list of genes between OXCT1 and SMIM5 genes (Fig. 4d, genes highlighted in red). It should be noted that two of the ER- fresh samples fell under the dendrogram arm of fresh ER+ samples, illustrating MammaPrint genes alone were not able to perfectly segregate cases based on ER status, even in fresh tissues. We were able to utilize the PAM50 published algorithms [4, 23] to evaluate the “intrinsic” molecular subtype as well as proliferative scores of ER+ and ER- samples in both FFPE and fresh RNA-seq data (Fig. 4d). Consistent with published literature [4, 38], we observed higher proliferation scores in ER- than ER+ samples within both FFPE and fresh tissue (Additional file 3: Figure S3a), confirming the utility of RNA-seq from archival FFPE breast cancer samples in these clinically relevant gene panels.

### Pathway validation of FFPE RNA-seq

**Fig. 5:**
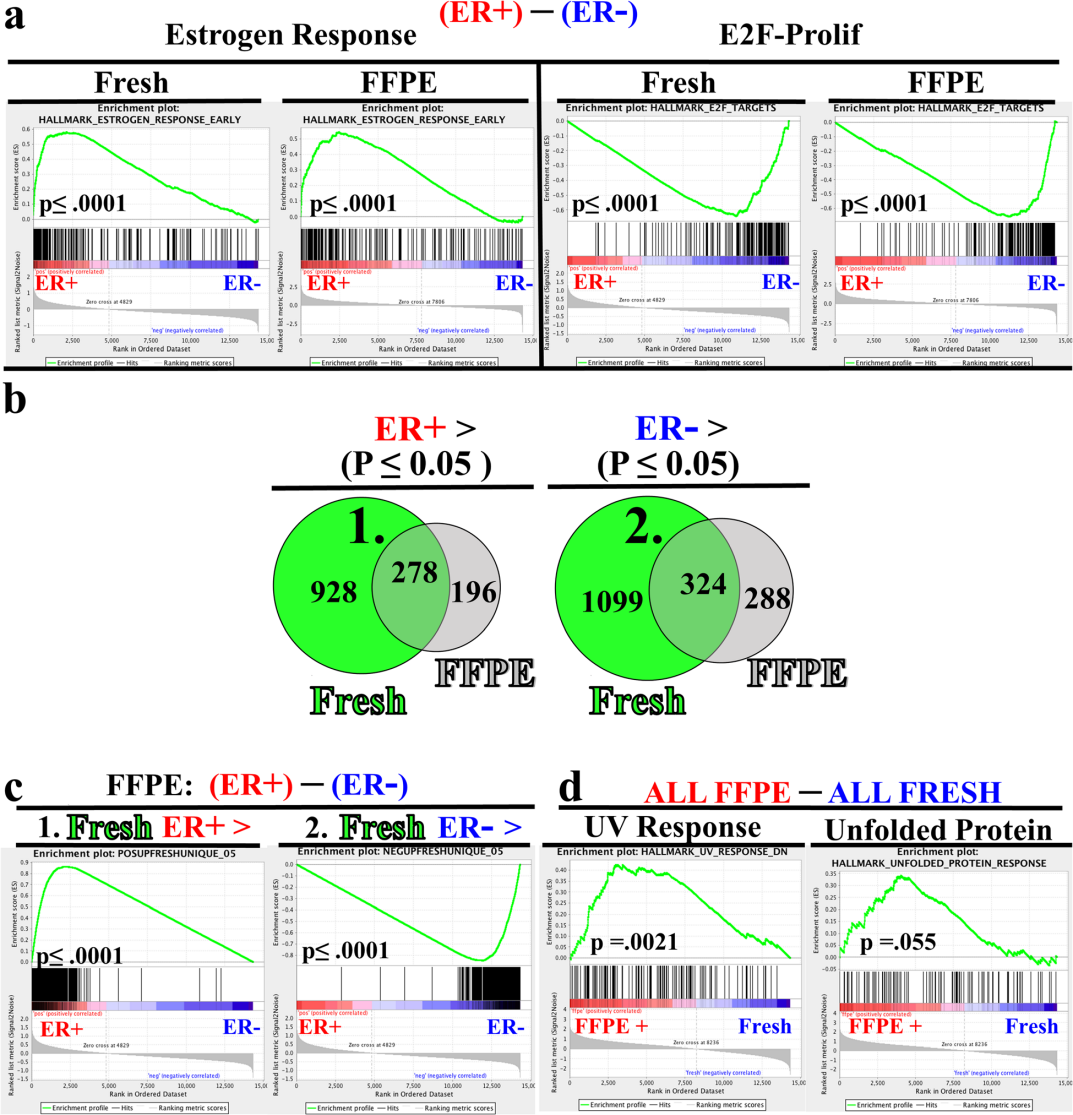
Pathway Validation of FFPE RNA-seq. Fresh and FFPE data sets were independently evaluated for differential gene expression based upon sample ER status. **a** Gene Set Enrichment Analysis (GSEA) determined significant enrichment for Estrogen Response genes (left) in ER+ samples and E2F-Proliferation associated genes in ER- samples for both FFPE and fresh specimens. **b** Venn-Diagram displaying overlapping and unique statistically significant differentially expressed genes (*p* ≤ 0.05, Fisher’s exact test using all input genes (*n =*14,330) as background results in *p* < 2.2e-16**)** between ER+ (red) and ER- (blue) samples from FFPE (gray) and fresh (green) specimens. Small numbers depict numbers of differentially expressed genes in the respective region. Shaded green represents genes identified in both FFPE and fresh data sets. **1.** and **2.** denote gene pools used for subsequent custom gene set analyses **c**) Gene set enrichment plots for FFPE samples derived from custom gene sets composed of 928 genes found significantly upregulated in ER+ fresh but not in FFPE samples, and 1099 genes significantly downregulated in ER+ fresh, but not in FFPE samples. **d** Hallmark GSEA enrichment plots of UV response and Unfolded Protein Response genes sets from analysis comparing all FFPE to all fresh specimens

Another powerful application for archival tissue is in the discovery of novel molecular biomarkers and pathways. To validate the use of FFPE tissue for discovery applications, we first compared performance of pathway analysis between ER+ and ER- samples separately for FFPE and fresh samples. For this analysis, we employed gene set enrichment analysis (GSEA) [39] utilizing the “Hallmark” gene pathways. In both fresh and FFPE samples (Fig. 5a, left), we observed highly significant enrichment of estrogen response genes in the ER+ (red) cases. Likewise, in both fresh and FFPE samples (Fig. 5a, right) we observed an enrichment of the proliferative E2F signature in the ER- (blue) samples. This analysis illustrates that two important, well-curated gene pathways remain intact in FFPE samples.

We next employed the common strategy of determining the overlap of individual genes that are significantly differentially expressed between two groups, in this case between FFPE and fresh frozen samples. We first compared gene lists of the differentially expressed genes between the ER+ and ER- samples from both FFPE and fresh frozen samples and observed 278 common statistically significant, differentially expressed genes upregulated in ER+ samples in both FFPE and fresh specimens (Fig. 5b, left shaded green). Likewise, we observed a similar number of common upregulated genes (324) in ER- samples (Fig. 5b, right shaded green), for a total of 602 total shared DE genes between FFPE and fresh groups. However, we also observed that approximately 3 times more genes were differentially expressed in the fresh group compared to the FFPE group (Fig. 5b). Thus, while GSEA analysis utilizing curated gene sets strongly suggested coherence between FFPE and fresh samples (Fig. 5a), this single-gene consideration of differential gene expression shows a reduced capacity to identify differentially expressed genes with FFPE samples.

To resolve the discordance between results from curated GSEA pathway analyses (Fig. 5a) and single-gene analysis (Fig. 5b, Venn Diagrams) we interrogated the FFPE data using custom gene sets rather than by individual genes. The first gene set was composed of genes significantly upregulated in ER+ fresh samples but not significant in FFPE samples (denoted by 1 (928 genes), Fig. 5b, left green). When considering this gene set as a whole, we found significant enrichment in FFPE ER+ samples (Fig. 5c, 1.- left panel). Likewise, the second custom gene set was composed of the genes most significantly overexpressed in ER- fresh samples but not found to be significant in FFPE samples (denoted by 2 (1099 genes), Fig. 5b, right green). Again, this gene set was also significantly enriched in the FFPE ER- samples (Fig. 5c, 2. right panel). These observations strongly argue that the biology that discriminates ER+ and ER- samples in fresh sections is highly conserved in FFPE specimens and is detected by FFPE RNA-seq when using gene set and rank-based analyses.

We also observed genes uniquely expressed in FFPE tissues (Fig. 5b, grey, and Additional file 3: Figure. S3b, shaded region). To broadly examine the idea of  uniquely expressed FFPE genes more deeply, we utilized GSEA with no consideration given to ER status, simply comparing all fresh samples to all FFPE samples. Gene sets related to UV response (Fig. 5d) and the Unfolded Protein Response (Fig. 5d) were significantly different between FFPE and fresh samples. Other stress response pathways that showed trending profiles included alternative gene sets for UV response and the P53 pathway (Additional file 3: Figure S3c). These observations are consistent with the hypothesis that some differential gene expression unique to FFPE tissues is driven by preservation and storage [7, 40]. While these stress response enrichment profiles are clearly stronger than differences observed between FFPE and fresh samples for ER- relevant hallmark gene sets (Additional file 3: Figure S3c, right two panels), we cannot conclusively determine the impact of tissue fixation and sample preservation, but rather offer a word of caution in considering stress response gene results from FFPE samples.

### FFPE pathway discovery through BrCa Regulon analysis

**Fig. 6:**
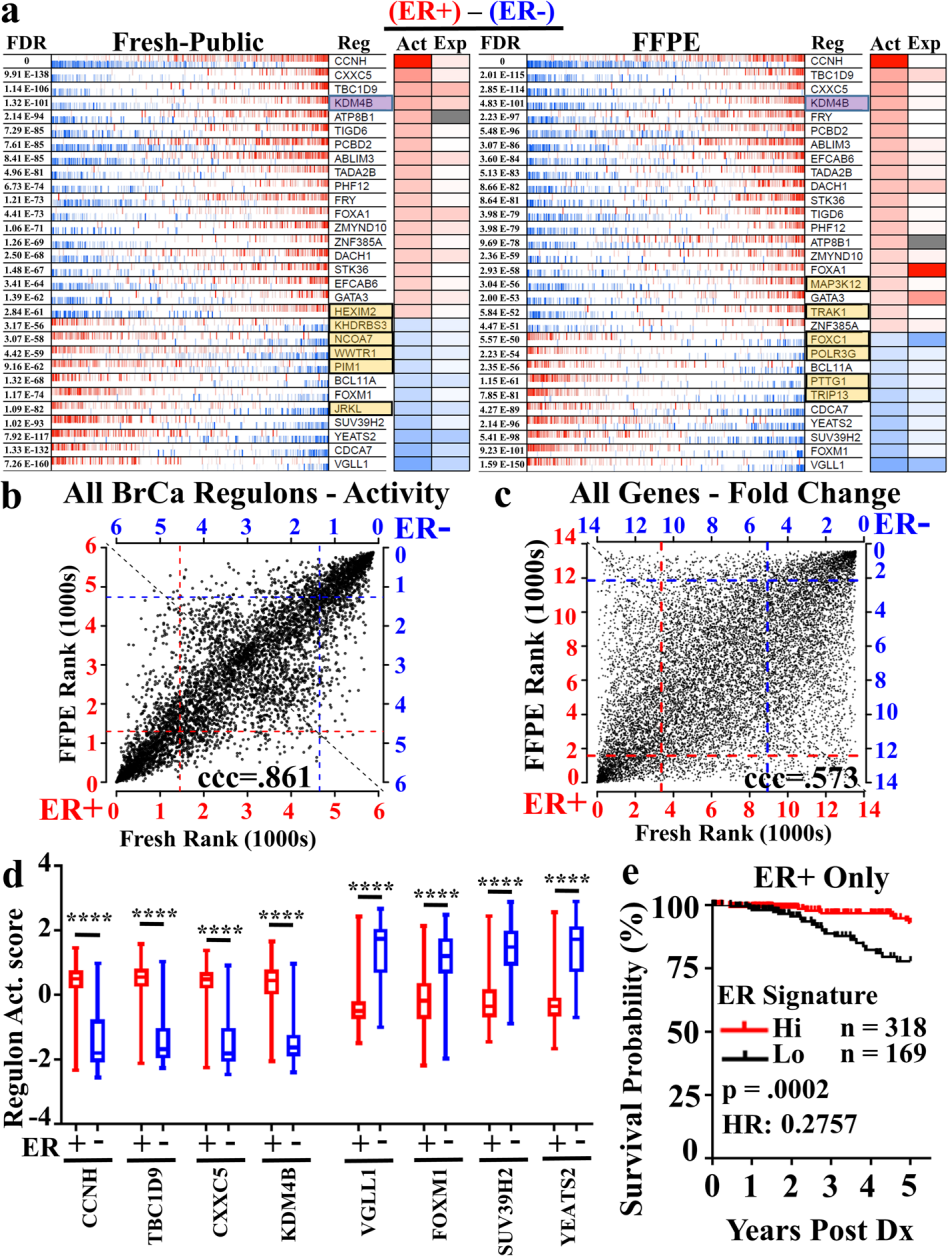
Regulon Validation of FFPE RNA-seq for pathway discovery reveals KDM4B as potential predictor of outcomes in ER+ TCGA breast cancer cohort. An established breast cancer regulon composed from The Cancer Genome Atlas (TCGA) specimens was utilized to interrogate differential signaling in transcription networks between ER+ and ER- samples from both FFPE and fresh specimen cohorts. **a** The 30 most differentially active regulons between ER+ and ER- samples in fresh (left) and FFPE (right) specimens are displayed. Shared regulons are shown in white boxes, and fresh vs. FFPE specific regulons are shown in orange. The activity (Act) of the regulon is displayed (red and blue for high and low network activity, respectively), as is the gene expression (Exp) of the transcription factor itself. **b** Plotting regulon (*n* = 5000) expression levels (Ranks) reveals high level of correlation between fresh and FFPE samples, whereas **c)** single-gene analysis (*n* = 14,330) reveals reduced correlation between fresh and FFPE. **d** Single-sample regulon activity scores were plotted in TCGA breast cancer cases annotated by IHC as ER+ (red, *n* = 478) or ER- (blue, *n* = 153) for each of the top 4 upregulated and downregulated regulons from the regulon analysis, depicting significant (****, *p* < .0001, Welch’s unpaired, two tailed, t-test) differences between ER+ and ER- samples and independently validating regulons results. **e** Five year survival probability within ER+ breast cancer patients stratified by highest (red, *n* = 318) and lowest (black, *n* = 169) ER Regulon Activity signature. ER Signature was composed by addition of z-score transformed single-sample regulon values as follows (KDM4B+CCNH-SUV39H2-YEATS2). *P*-value determined by log-rank test and gene expression data retrieved and filtered by UCSC Xena Functional Genomics Explorer

We next considered newer computational approaches employing unbiased, in-silico constructed gene association networks known as regulons [24, 25]. Regulon analysis utilizes tightly correlated alterations in genes and known transcription factor/regulator [26] expression to generate transcription factor/gene networks (regulons) in an unbiased manner. Similar to Gene Set Enrichment Analysis, differential activity of the whole network, rather than the performance of individual genes, determines pathway relevance. We utilized a breast cancer regulatory network previously established from the evaluation of hundreds TCGA breast cancer samples [27], and applied this analysis to our ER+ and ER- cohorts of fresh and FFPE samples separately (Fig. 6a). When we evaluated the top 30 most active regulons (Fig. 6a) between ER+ and ER- samples in fresh (left) and FFPE (right) samples, we observed 24 of the top 30 networks (80%) were conserved, while 6 (highlighted in orange) of the top 30 for each were unique to either FFPE or fresh samples.

To further illustrate regulon conservation between fresh and FFPE samples, we plotted the rank of the activity score for all regulons (~ 5000) in FFPE by their rank in fresh (Fig. 6b). In this analysis, we observed a strong 1:1 linear correlation (ccc =0.861) of up- and down-regulated regulons between FFPE and fresh tissue. This strong correlation contrasted greatly with the significantly reduced concordance observed when we examined the relative rank of differential expression of individual genes (ccc = 0.573, Fig. 6c). This formal demonstration of a great degree of conservation in transcription network biology between the fresh and FFPE samples compared to single-gene analyses strongly supports the adoption of regulon analysis for FFPE data sets.

To further validate our FFPE-derived regulon gene expression signatures, we explored if these regulons could delineate ER+ and ER- breast cancer using the TCGA dataset, where RNA expression data is obtained from fresh tissue. We selected all 897 female primary breast cancer patients under the age of 76, and subset the cases based upon clinically annotated (IHC) ER status, identifying 478 ER+ and 153 ER- cases. Next, for the four most up-regulated and four most down-regulated FFPE regulons (Fig. 6a), single-sample regulon activity scores were plotted demonstrating highly statistically significant differences (*p* < .0001) between ER+ and ER- cohorts (Fig. 6d). Further, the expression values for each transcription factor defining each regulon similarly discriminated ER+ and ER- cohorts (Additional file 4: Figure. S4a,b). These data reinforce the fidelity of the FFPE regulon results.

Since ER+ and ER- breast cancers differ in 5 year survival rates [41, 42], we next determined if the 8 FFPE-derived regulons provide improved correlation with patient outcomes compared to single-gene analysis. Seven out of the 8 regulons identified in our FFPE regulon results showed statistical significance in 5 year overall survival between the high and low cohort compared to only 4 out of 8 single-gene analyses (Table 2). Of the 8 regulons, only the VGLL1 regulon activity (and gene expression), although clearly differentially associated with ER status (Fig. 6d, Additional file 4: Figure S4a), showed no survival prognostic value in the TCGA cohort (Fig. 6d, Additional file 4: Figure S4a). Lastly, the histone demethylase KDM4B is the ER upregulated pathway with the strongest prognostic indicator of survival in both single-regulon and single-gene analyses.

**Table 2:** 5 year Overall Survival in TCGA breast cancer cohort based upon regulon activity or gene expression

TCGA Breast Cancer: *n* = 897		Single Sample Regulon Activity			Single Sample Gene Expression Value		
Regulon	Gene	HR (Hi/Lo)	*p* value (Log-Rank)	End survival % (Hi/Lo)	HR (Hi/Lo)	*p* value (Log-Rank)	End Survival % (Hi/Lo)
CCNH		0.3864	0.0001	90/78	0.6962	0.1258	86/82
TBC1D9		0.4976	0.0043	89/80	0.4555	0.0014	89/79
CXXC5		0.4641	0.002	90/79	0.7015	0.1409	86/82
KDM4B		0.3221	<.0001	92/77	0.3786	<.0001	90/78
VGLL1		1.108	0.663	85/84	1.46	0.1114	82/86
FOXM1		2.065	0.0029	80/89	1.993	0.0044	80/89
SUV39H2		2.929	<.0001	77/92	2.279	0.0009	79/90
YEATS2		3.434	<.0001	76/92	1.429	0.1292	82/87

RNA expression data from 897 female primary breast cancer patients under the age of 76 was obtained from the TCGA cohort and stratified to either the high (Hi) or low (Lo) group for each of the individual indicated regulons or genes based upon median values for regulon activity or gene expression (depicted in Additional file 4: Figure S4b). Five year overall survival distribution was determined and Hazard Ratio (HR) expressed as risk comparing the high (Hi) expression/activity to the low (Lo) group. Log-rank *p*-values are displayed and the final frequency of survival at 5 years post diagnosis indicated for the high and low groups (Hi/Lo)

To extend the application of our FFPE regulon results, we set out to establish an optimized ER activity score based upon the selective combination of single regulon activities. We reasoned that gene networks that were up- vs. down-regulated in response to ER activity likely represent different sets of genes, so to perform this analysis we explored the impact of combining the significantly up-regulated with the significantly down-regulated regulons. We evaluated the biological significance of three signature combinations by distributing all 897 breast cancer cases into high- or low-expressing groups and evaluating survival (Additional file 5: Table S1, Additional file 4: Figure S4c). Not surprisingly, the addition of more than one regulon activity score enhanced the stratification of outcomes in the entire TCGA dataset, with optimal gains occurring with the following signature CCNH+KDM4B-SUV39H2-YEATS2 (Additional file 5: Table S1, Additional file 4: Figure S4d). We then applied this ER activity signature to only clinically-annotated ER+ samples. We observed approximately 1/3 of all clinically-annotated ER+ breast cancer cases displayed a low ER activity signature, which correlated with poor 5 year overall survival (Fig. 6e). Importantly, we could not delineate good and poor prognostic ER+ cases using the highest and lowest quartiles of single-gene expression data for the estrogen receptor (ESR1, log-rank *p* = .9060) or progesterone receptor (PGR, log-rank *p* = .08452, 5 year ER+ cohort overall survival depicted in Additional file 4: Figure S4e). These data illustrate a successful implementation of regulon transcription network analysis in FFPE tissues for elucidating novel discoveries.

## Discussion

FFPE samples represent a unique source of biological information. The samples themselves are a snapshot of a patient’s biology that remains frozen in time while the patient goes on to experience disease outcomes and response to treatment. FFPE tissues therefore represent a resource with inherently increasing value as outcomes data mature. This unique, clinically relevant value however comes at a cost of damaged RNA and DNA molecules from fixation and storage processes. Nonetheless, molecular interrogation of these resources for biological insight has been performed by RT-PCR for both RNA and DNA. These studies have demonstrated that through limited and selective generation of complementary oligonucleotides, RNA from FFPE tissues are generally suitable for both diagnostic nucleic acid hybridization assays and PCR use [15, 16, 32, 37, 43].

With Next-Generation Sequencing technology, both whole genome and whole exome profiling of fresh patient samples has led to deeper patient stratification and cancer subtype allocation, representing important advances in personalized medicine. For FFPE tissues however, this technology operates outside of the targeted and validated oligo approach that has enabled confident use of FFPE tissues for evaluation of RNA expression. While FFPE RNA-seq has been increasingly employed in recent years to stratify patient cohorts based upon global gene expression data [44–49], far fewer reports have made extensive efforts to address the impact of known artifacts of tissue preservation on the fidelity of RNA expression profiles through thorough comparisons with fresh tissue [9, 10, 30, 45, 47, 50–59]. Even within these existing reports there remain limitations in interpreting the level of fidelity provided by FFPE tissue through FFPE RNA-seq. These limitations include: comparisons between matched fresh frozen tissue with non-long-term archived FFPE tissue [10, 50, 51, 56], examining only a limited set of genes [58–61], use of high intra-variant cohorts [54], focus on single-sample analyses [30, 53, 62], exclusive focus on cohort level difference [52, 56] and narrowed evaluation of technical sequencing metrics [9, 10, 13, 55].

Our approach has been to adopt a true-to-research workflow by using repository archival tissues complemented with public data from fresh cohorts, resources routinely available to basic science researchers. In our approach, the systematic employment of bioinformatics revealed high concordance between fresh and FFPE gene signatures, enabling successful biomarker discovery despite the fact that the cohorts of FFPE and fresh tissues utilized were not subject-matched, prepared by the same library method, nor sequenced by the same facility. This success was highly dependent upon the targeted employment of results from pathway analyses rather than reliance upon single-gene profiles. The coherence between FFPE and fresh tissues, as well as the demonstrated significance in outcomes, endorses the confident utilization of clinically annotated FFPE specimens for expression profiling work.

While more accessible and abundant than fresh tissue, FFPE still represents a limited resource that requires thoughtful utilization. We importantly illustrate in this report that we can obtain reliable gene expression information of 14,000+ genes from a single 10 μm section of FFPE breast cancer tissue. This finding represents a dramatic improvement in the number of molecular targets that can be evaluated from relatively modest amounts of FFPE tissue. We also report a high failure rate in FFPE tissues to achieve sufficient quality RNA. In our study of 58 cases, the large failure rate for obtaining suitable RNA samples (DV200 ≥ 30), was observed to be largely dependent upon the age of the block, but not in a predictably linear fashion. Rather than age alone, our data may reflect shifts in tissue collection or preservation practices, which warrants further investigation. In addition to providing optimized recommendations for the amount of tissue required and block age, we also describe the impact of library selection on expression results. In agreement with previous studies, our study reveals that library preparation introduces the greatest level of variation, greater than that observed between FFPE and fresh tissue cohorts. Previously, others have demonstrated that variance in ribosomal depletion strategies, or selection of either Poly A and/or targeted capture oligo bead hybridization libraries, all differentially change count and relative abundance results that impact evaluation of global gene expression concordance [10, 12, 51]. Based on these results, we suggest that library selection be driven by the underlying experimental question.

We have also shown and validated by comparison to fresh tissues, to our knowledge, the first successful application of RNA-seq regulon analysis for FFPE specimens. Evaluation of regulon activity can overcome many of the barriers imposed by analysis of single-gene expression levels, which can be significantly impacted by tissue preservation approaches. This observation is in line with more recent analytic approaches such as relative gene ranking [57] or reiterative revision of established gene sets (GSEA, etc.) to more faithfully characterize tissue cellular composition (immune and epithelial cell frequency) and cellular processes (cell cycling) from bulk FFPE tissues [11]. Of the 8 regulons identified to be differentially expressed between ER+ and ER- FFPE breast cancer cases, the combination of 4 regulons CCNH+KDM4B-SUV39H2-YEATS2 maximally delineated outcomes in the TCGA breast cancer dataset. Of these four regulons, the regulon defined by the histone demethylase gene KDM4B, an ERassociated transcriptional regulator [63, 64], best informed overall survival probability in ER+ cases, whereas ESR1 or PGR did not. One hypothesis is that this result stems from KDM4B expression being a more faithful indicator of active estrogen receptor signaling than either ESR1 or PGR expression, and therefore identifies patients most likely to have benefited from hormone deprivation therapy. While future investigations are required to establish the connection between KDM4B and ER+ breast cancer survival, these results illustrate a successful utilization of true archival FFPE tissues for potentially clinically relevant biomarker identification by RNA-seq and is, to our knowledge, the first report of differential expression and activity of KDM4B within ER+ breast cancers correlating to patient survival.

## Conclusion

In sum, our FFPE RNA-seq pipeline supports the approach of utilizing clinically-annotated FFPE cancer tissues with outcomes data to address key questions in the breast cancer field, including the delineation between indolent and life-threatening disease and the molecular mechanisms of treatment resistance. Additionally, investigation of archival FFPE tissues with treatment and outcomes data through global RNA-seq profiling could be utilized to enhance the performance of prognostic gene panels, increasing their utility for patient treatment stratification. Further, in the exploding era of bioinformatics and 'omic analytics, new tools such as CITE-Seq and cellular deconvolution [65] will further enhance and refine our understanding of RNA signatures obtained from FFPE tissue [66]. We encourage researchers in these fields to incorporate FFPE tissues into their studies, as an easily obtainable, faithful and valuable resource for RNA expression signatures.

## Supplementary information

**Additional file 1: Figure S1.** Repeatability of RNA assessment. RNA isolated from 6 FFPE samples were assessed at separate times (run) to evaluate variance in determination of **a)** yield from Nanodrop and Bioanalyzer instruments and **b)** RNA quality by evaluation of DV200 values. Each separate specimen is identified by a different colored symbol with values corresponding to matched samples across runs connected by the line. **c)** Plots of unique reads as % of total reads and **d)** overall number of unique reads in Access (Acc) vs. Ovation (Ova) RNA library preparation kits for sample 1 (orange) and sample 2 (green). Limma normalization of gene expression in Access (Acc) vs. Ovation (Ova) libraries for both sample 1 (orange and red, in triplicate) and sample 2 (green and purple, in triplicate), results in **e)** linear relationship across the range with raw values and **f)** count bias toward the Acc library.

**Additional file 2: Figure S2.** Extended evaluation of global gene expression reveals high concordance between FFPE and fresh samples. a) Sequence alignment parameters of clipping (no = light blue, soft = light purple) and allowed nucleotide mismatches (mm) of 3–15 were evaluated for impact of aligned read percentage for the 6 FFPE samples in comparison to soft-clipping utilization in fresh (Fr, *n* = 20) samples. **b)** Global linear correlation evaluation of FFPE compared to fresh samples based upon the average gene expression determined in Fresh plotted by the average gene expression determined in FFPE in ER+ (left) and ER-(right) cases. The best fit linear line is depicted in red from which r values were derived.

**Additional file 3: Figure S3.** FFPE compared to fresh samples utilizing the PAM50 genes and GSEA. a) Gene expression values from genes included in the PAM50 gene panel (Fig. 4d) were utilized to assign PAM50 Proliferation, ER and HER2 predictions scores for *n* = 6 FFPE (left panel) and n = 20 fresh cases (right panel) specimens. The scores reflect similar performance based upon ER subtype (ER + =red, ER- = blue), independent of tissue processing. **b)** Plot of adjusted *p*-value and log 2 fold change comparing all genes between FFPE and fresh specimens, regardless of ER status. Shading highlights genes distinctly unique to each group. **c)** GSEA enrichment profiles comparing all FFPE samples (red) to all fresh samples (blue), demonstrating trending enrichment in UV response and P53 gene sets (left two panels) in FFPE samples while no enrichment is observed in E2F and Estrogen Response (right two) gene sets for either sample type.

**Additional file 4: Figure S4.** Validation of ER+ vs. ER- regulon results from FFPE tissues. From the TCGA cohort, RNA expression data from 478 ER+ (blue) and 153 ER– negative (red) primary breast cancer tumors from women under the age of 76 were evaluated for **a)** single-gene expression of the most differentially active regulon factors from Fig. 6, illustrating highly statistically significant differences between ER+ and ER- samples (****, *p* < .0001, Welch’s unpaired, two tailed, t-test) and **b)** plotted for single-sample gene expression by regulon activity score. Dashed lines represent median values based upon the entire 897 TCGA breast cancer cohort used for identifying high and low expression (Table 2). Optimal ER activity regulon signature was empirically determined (Additional file 5**: Table S1**) for separation of 5 year Overall Survival (O.S.) probability post-diagnosis (Dx) in the 897 TCGA primary female breast cancer cohort under the age of 76. Distribution of this score **c)** was plotted for all cases (gray, *n* = 897) or TCGA-annotated ER+ (red, *n* = 478) or ER– (blue, *n* = 153) cases. Dotted line depicts threshold value for Hi vs. Lo designation based upon whole cohort distribution. **d)** Overall Survival (O.S.) probability is depicted for all the included TCGA breast cancer cases based upon Hi (*n* = 473) vs. Lo (*n* = 424) optimized ER signature score, revealing highly statistically significant differences (*p* < .0001, log-rank) with a Hazard Ratio (HR) favoring 5 year survival of ER signature high patients. **e)** 5 year Overall Survival probability of ER+ (*n* = 478) breast cancer patients

**Additional file 5: Table S1**. ER Regulon Activity Signature determined 5 year Overall Survival in TCGA breast cancer cohort or ER+ only cases. Single sample regulon activity values were z-score transformed based upon data from all 897 female primary breast cancer patients under the age of 76 obtained from the TCGA. Z-score regulon activity values were then selected and combined based upon the significance in impacting survival as determined in Supplemental Table 1. The indicated 3 ER activity regulon signature values were then used to stratify to samples to either high (Hi) or low (Lo) groups based upon the distribution of the whole cohort. 5 year overall survival distribution was determined and Hazard Ratio (HR) expressed as risk comparing the high (Hi) expression/activity to low (Lo) group for all TCGA cases (left, *n* = 897) or for just IHC-annotated ER+ cases (right, *n* = 478). Log-rank *p*-values are displayed and the final frequency of survival at 5 years post diagnosis indicated for the high and low groups (Hi/Lo) for all cases and ER+ only cases separately.

## Data Availability

The RNA-seq datasets generated and/or used during the current study have been deposited and are available from Gene Expression Omnibus (GEO) repository with the accession code GSE130397 https://www.ncbi.nlm.nih.gov/geo/query/acc.cgi?acc=GSE130397, or otherwise available from the corresponding author upon reasonable request.

## Abbreviations

BrCa: Breast Cancer
CV: Coefficient of variation
DE: Differentially expressed
DEG: Differentially expressed gene(s)
Dx: Diagnosis
ER/ESR: Estrogen Receptor
FFPE: Formalin-fixed, paraffin-embedded
GSEA: Gene set enrichment analysis
IHC: Immunohistochemistry
NMF: Non-negative matrix factorization
O.S.: Overall Survival
OHSU: Oregon Health & Science University
PCA: Principal Component Analysis
PCR: Polymerase chain reaction
PGR: Progesterone receptor
TCGA: The Cancer Genome Atlas
UV: Ultraviolet
VST: Variance stabilizing transformation
