# Supplementary figures and images for: RNA-seq from archival FFPE breast cancer samples: molecular pathway fidelity and novel discovery
Source: BMC Med Genomics. 2019 Dec 19;12:195. doi: 10.1186/s12920-019-0643-z (PMC6924022; doi:10.1186/s12920-019-0643-z)

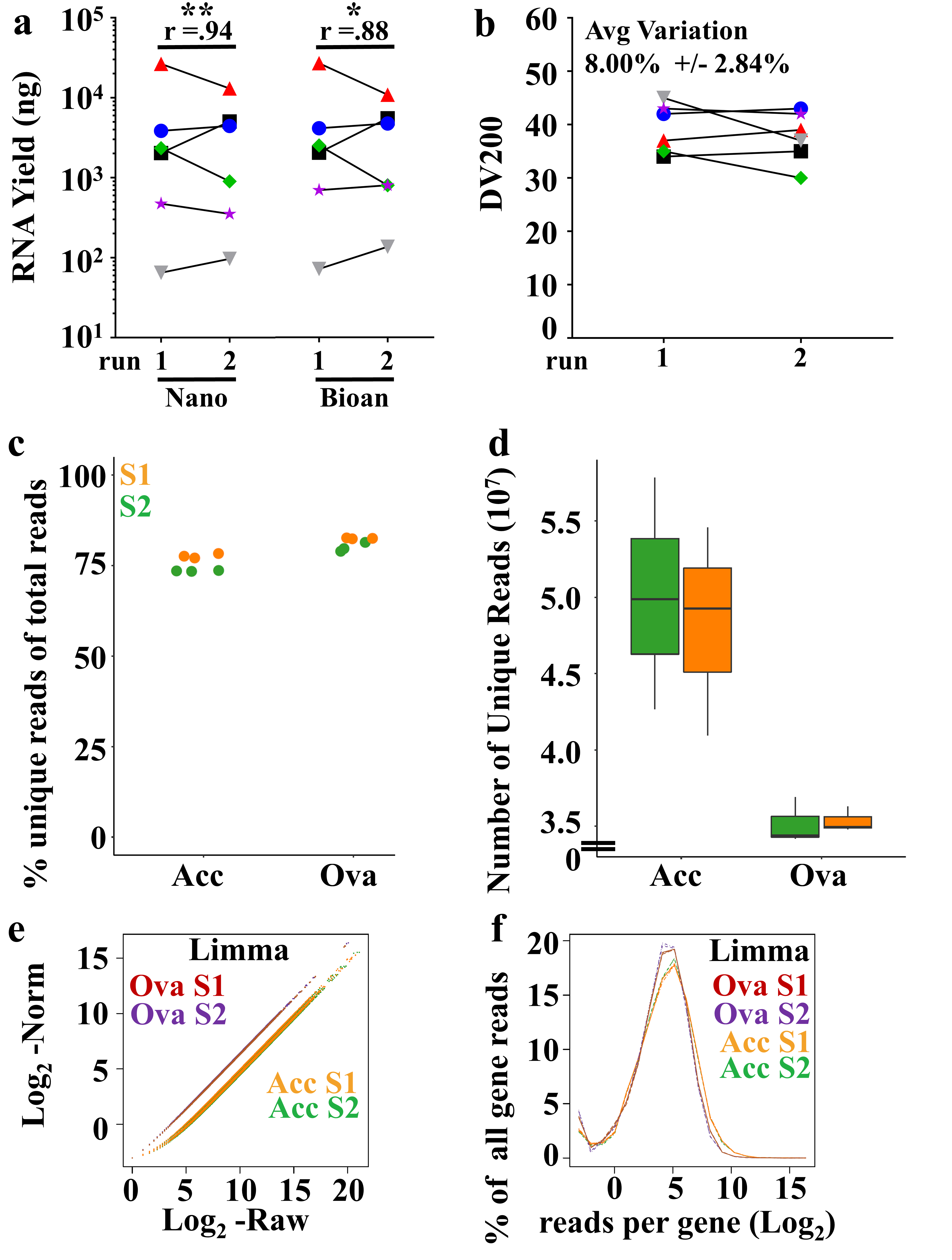

Supplement: Supplementary file 1 — Additional file 1: Figure S1. Repeatability of RNA assessment. RNA isolated from 6 FFPE samples were assessed at separate times (run) to evaluate variance in determination of a) yield from Nanodrop and Bioanalyzer instruments and b) RNA quality by evaluation of DV200 values. Each separate specimen is identified by a different colored symbol with values corresponding to matched samples across runs connected by the line. c) Plots of unique reads as % of total reads and d) overall number of unique reads in Access (Acc) vs. Ovation (Ova) RNA library preparation kits for sample 1 (orange) and sample 2 (green). Limma normalization of gene expression in Access (Acc) vs. Ovation (Ova) libraries for both sample 1 (orange and red, in triplicate) and sample 2 (green and purple, in triplicate), results in e) linear relationship across the range with raw values and f) count bias toward the Acc library. [file 12920_2019_643_MOESM1_ESM.tif]

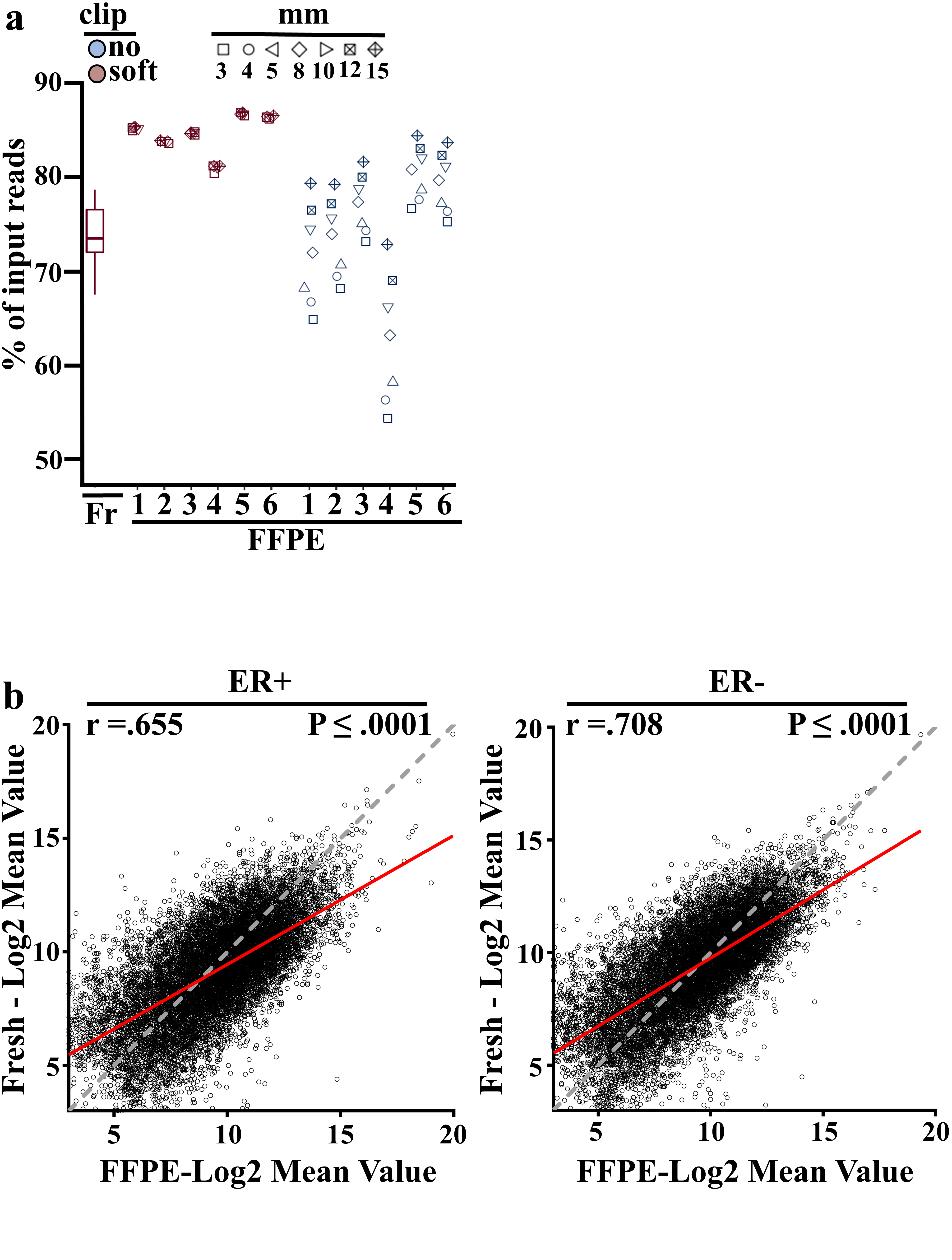

Supplement: Supplementary file 2 — Additional file 2: Figure S2. Extended evaluation of global gene expression reveals high concordance between FFPE and fresh samples. a) Sequence alignment parameters of clipping (no = light blue, soft = light purple) and allowed nucleotide mismatches (mm) of 3–15 were evaluated for impact of aligned read percentage for the 6 FFPE samples in comparison to soft-clipping utilization in fresh (Fr, n = 20) samples. b) Global linear correlation evaluation of FFPE compared to fresh samples based upon the average gene expression determined in Fresh plotted by the average gene expression determined in FFPE in ER+ (left) and ER-(right) cases. The best fit linear line is depicted in red from which r values were derived. [file 12920_2019_643_MOESM2_ESM.tif]

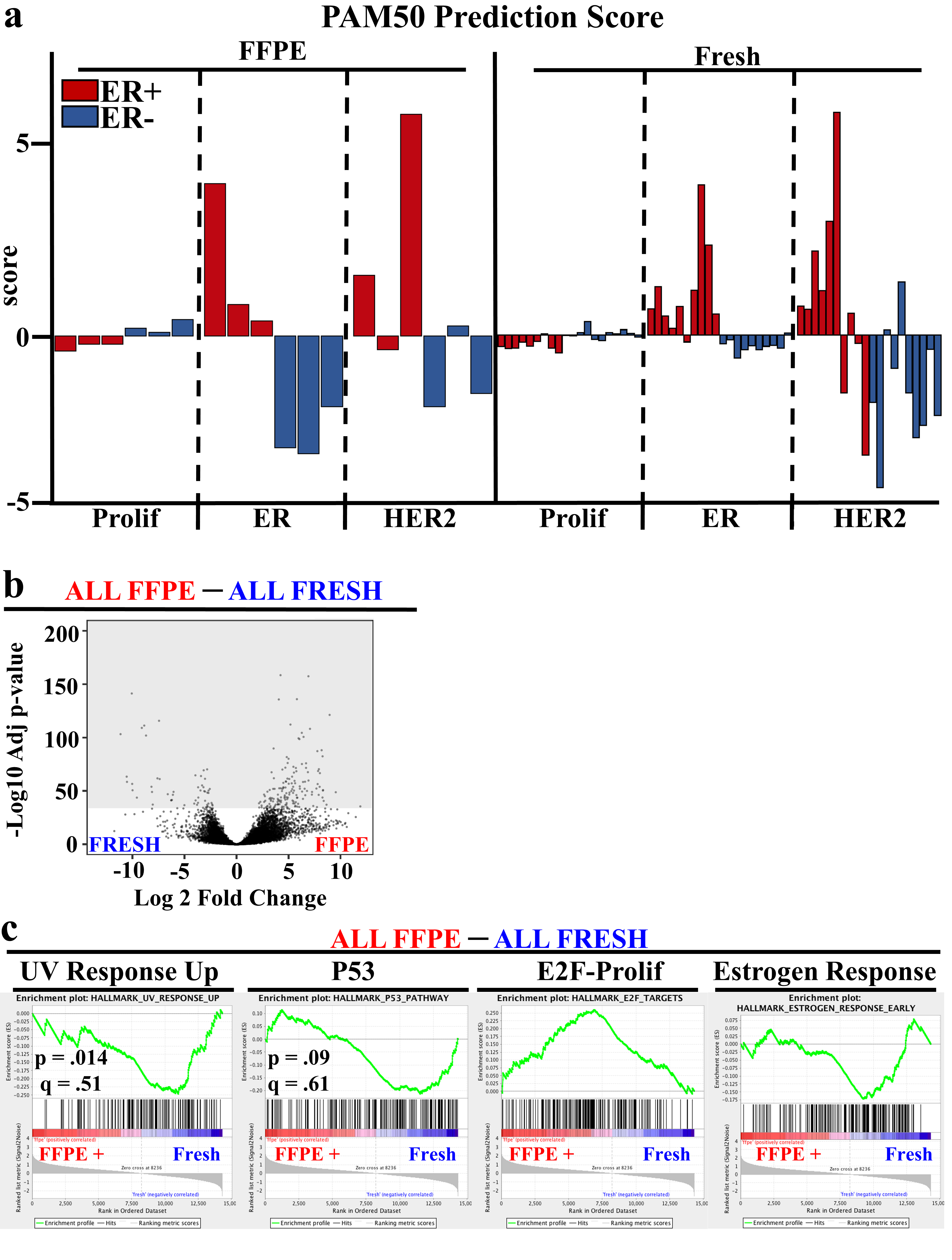

Supplement: Supplementary file 3 — Additional file 3: Figure S3. FFPE compared to fresh samples utilizing the PAM50 genes and GSEA. a) Gene expression values from genes included in the PAM50 gene panel (Fig. 4d) were utilized to assign PAM50 Proliferation, ER and HER2 predictions scores for n = 6 FFPE (left panel) and n = 20 fresh cases (right panel) specimens. The scores reflect similar performance based upon ER subtype (ER + =red, ER- = blue), independent of tissue processing. b) Plot of adjusted p-value and log 2 fold change comparing all genes between FFPE and fresh specimens, regardless of ER status. Shading highlights genes distinctly unique to each group. c) GSEA enrichment profiles comparing all FFPE samples (red) to all fresh samples (blue), demonstrating trending enrichment in UV response and P53 gene sets (left two panels) in FFPE samples while no enrichment is observed in E2F and Estrogen Response (right two) gene sets for either sample type. [file 12920_2019_643_MOESM3_ESM.tif]

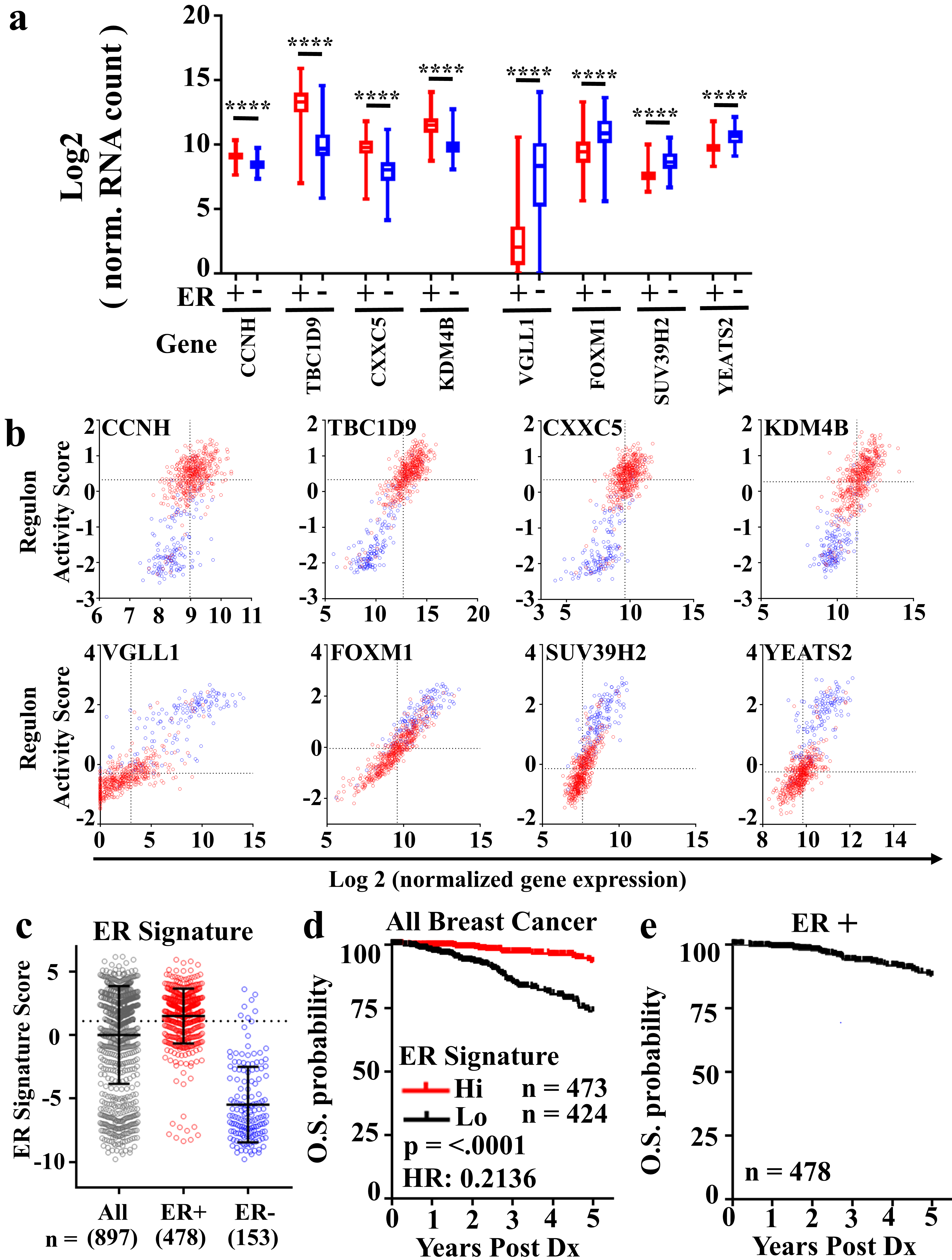

Supplement: Supplementary file 4 — Additional file 4: Figure S4. Validation of ER+ vs. ER- regulon results from FFPE tissues. From the TCGA cohort, RNA expression data from 478 ER+ (blue) and 153 ER– negative (red) primary breast cancer tumors from women under the age of 76 were evaluated for a) single-gene expression of the most differentially active regulon factors from Fig. 6, illustrating highly statistically significant differences between ER+ and ER- samples (****, p < .0001, Welch’s unpaired, two tailed, t-test) and b) plotted for single-sample gene expression by regulon activity score. Dashed lines represent median values based upon the entire 897 TCGA breast cancer cohort used for identifying high and low expression (Table 2). Optimal ER activity regulon signature was empirically determined (Additional file 5: Table S1) for separation of 5 year Overall Survival (O.S.) probability post-diagnosis (Dx) in the 897 TCGA primary female breast cancer cohort under the age of 76. Distribution of this score c) was plotted for all cases (gray, n = 897) or TCGA-annotated ER+ (red, n = 478) or ER– (blue, n = 153) cases. Dotted line depicts threshold value for Hi vs. Lo designation based upon whole cohort distribution. d) Overall Survival (O.S.) probability is depicted for all the included TCGA breast cancer cases based upon Hi (n = 473) vs. Lo (n = 424) optimized ER signature score, revealing highly statistically significant differences (p < .0001, log-rank) with a Hazard Ratio (HR) favoring 5 year survival of ER signature high patients. e) 5 year Overall Survival probability of ER+ (n = 478) breast cancer patients [file 12920_2019_643_MOESM4_ESM.tif]
