# Supplementary material for: RNA-seq from archival FFPE breast cancer samples: molecular pathway fidelity and novel discovery
Source: BMC Med Genomics. 2019 Dec 19;12:195. doi: 10.1186/s12920-019-0643-z (PMC6924022; doi:10.1186/s12920-019-0643-z)
Supplement: Supplementary file 5 — Additional file 5: Table S1. ER Regulon Activity Signature determined 5 year Overall Survival in TCGA breast cancer cohort or ER+ only cases. Single sample regulon activity values were z-score transformed based upon data from all 897 female primary breast cancer patients under the age of 76 obtained from the TCGA. Z-score regulon activity values were then selected and combined based upon the significance in impacting survival as determined in Supplemental Table 1. The indicated 3 ER activity regulon signature values were then used to stratify to samples to either high (Hi) or low (Lo) groups based upon the distribution of the whole cohort. 5 year overall survival distribution was determined and Hazard Ratio (HR) expressed as risk comparing the high (Hi) expression/activity to low (Lo) group for all TCGA cases (left, n = 897) or for just IHC-annotated ER+ cases (right, n = 478). Log-rank p-values are displayed and the final frequency of survival at 5 years post diagnosis indicated for the high and low groups (Hi/Lo) for all cases and ER+ only cases separately. [file 12920_2019_643_MOESM5_ESM.docx]

**Supplemental Table 1**

| **Supplemental Table 1:**  **ER Regulon Signature Outcomes** | **TCGA All Breast Cancer**  **n= 897** | | | **Annotated ER+ Breast Cancer Only**  **n = 478** | | |
| --- | --- | --- | --- | --- | --- | --- |
| **Regulon Signature** | **HR (Hi/Lo)** | **p value (Log-Rank)** | **End Survival % (Hi/Lo)** | **HR (Hi/Lo)** | **p value (Log-Rank)** | **End Survival % (Hi/Lo)** |
| **4 Up - 3 Down (CCNH+TBC1D9+CXXC5+ KDM4B) - (FOXM1+ SUV39H2 + YEATS2)** | 0.2223 | <.0001 | 94/74 | 0.3048 | 0.0006 | 93/79 |
| **2 Up - 2 Down (CCNH+ KDM4B) –**  **( SUV39H2 + YEATS2)** | 0.2136 | <.0001 | 94/74 | 0.2757 | 0.0002 | 94/78 |
| **1 Up - 1 Down KDM4B - YEATS2** | 0.2379 | <.0001 | 93/75 | 0.2919 | 0.0005 | 94/79 |
